# The Innate Immune Response of Atlantic Salmon (*Salmo salar*) Is Not Negatively Affected by High Temperature and Moderate Hypoxia

**DOI:** 10.3389/fimmu.2020.01009

**Published:** 2020-05-27

**Authors:** Fábio S. Zanuzzo, Anne Beemelmanns, Jennifer R. Hall, Matthew L. Rise, Anthony K. Gamperl

**Affiliations:** ^1^Department of Ocean Sciences, Memorial University, St. John's, NL, Canada; ^2^Aquatic Research Cluster, CREAIT Network, Ocean Sciences Centre, Memorial University, St. John's, NL, Canada

**Keywords:** aquaculture, global warming, fish, disease susceptibility, immune response, climate change, high temperature, hypoxia

## Abstract

Climate change is predicted to increase water temperatures and decrease oxygen levels in freshwater and marine environments, however, there is conflicting information regarding the extent to which these conditions may impact the immune defenses of fish. In this study, Atlantic salmon were exposed to: (1) normoxia (100–110% air saturation) at 12°C; (2) an incremental temperature increase (1°C per week from 12 to 20°C), and then held at 20°C for an additional 4 weeks; and (3) “2” with the addition of moderate hypoxia (~65–75% air saturation). These conditions realistically reflect what farmed salmon in some locations are currently facing, and future conditions in Atlantic Canada and Europe, during the summer months. The salmon were sampled for the measurement of head kidney constitutive anti-bacterial and anti-viral transcript expression levels, and blood parameters of humoral immune function. Thereafter, they were injected with either the multi-valent vaccine Forte V II (contains both bacterial and viral antigens) or PBS (phosphate-buffer-saline), and the head kidney and blood of these fish were sampled at 6, 12, 24, and 48 h post-injection (HPI). Our results showed that: (1) neither high temperature, nor high temperature + moderate hypoxia, adversely affected respiratory burst, complement activity or lysozyme concentration; (2) the constitutive transcript expression levels of the anti-bacterial genes *il1*β, *il8-a, cox2, hamp-a, stlr5-a*, and *irf7-b* were up-regulated by high temperature; (3) while high temperature hastened the peak in transcript expression levels of most anti-bacterial genes by 6–12 h following V II injection, it did not affect the magnitude of changes in transcript expression; (4) anti-viral (*viperin-b, mx-b*, and *isg15-a*) transcript expression levels were either unaffected, or downregulated, by acclimation temperature or V II injection over the 48 HPI; and (5) hypoxia, in addition to high temperature, did not impact immune transcript expression. In conclusion, temperatures up to 20°C, and moderate hypoxia, do not impair the capacity of the Atlantic salmon's innate immune system to respond to bacterial antigens. These findings are surprising, and highlight the salmon's capacity to mount robust innate immune responses (i.e., similar to control fish under optimal conditions) under conditions approaching their upper thermal limit.

## Introduction

According to the Intergovernmental Panel on Climate Change Assessment report of 2014 and the special report of 2019 (1), the surface temperature of the world's oceans is expected to increase by 1–3°C over the next 80 years [also see Collins et al. (2)]. Therefore, understanding how elevated temperatures affect marine species is critical, and a growing focus worldwide (3–6). In addition, reductions in water dissolved oxygen content (hypoxia) are expected to impact the health and distribution of marine organisms as the frequency and severity of hypoxic events are also increasing (7–9). Marine aquaculture sites are particularly vulnerable to changes in these environmental conditions, as they are generally in coastal regions, and the fish are confined and cannot seek out conditions that might be more favorable / optimal. For example, temperatures have recently exceeded 22–23°C in Atlantic salmon (*Salmo salar*) sea-cages in Tasmania, water temperatures often reach 18–20°C during the summer in Canada and Norway, and it is not uncommon for oxygen levels to drop below 70% of air saturation during these periods (10–12). These changes in environmental conditions represent a considerable challenge for the aquaculture industry as they can affect the growth, health and welfare of fish held in sea-cages [e.g., see Wade et al. (13)].

A properly functioning immune system is key to the ability of fish to resist infection by bacteria and viruses, and it is well established that water temperature modulates fish immune function (14–16). However, there is contradicting information regarding the impact(s) of high temperature on the ability of fish to mount an effective defense against pathogens [e.g., see Makrinos and Bowden (15)]. For example, while several studies have reported that high temperatures have a beneficial effect (17–24), a number of studies have demonstrated a relatively small effect (25–27), the opposite effect (19, 28–32), or conflicting and complex results (33, 34). The reasons for these disparate results may be, in part, related to the rate of temperature increase (i.e., acute vs. chronic), the range of temperatures used (i.e., whether they exceeded the physiological/optimum range for that particular species), as well as the particularities/complexity of the teleost immune system; i.e., some fish appear to rely more on non-specific (innate) responses at low temperatures, whereas at higher temperatures they may depend more on specific (acquired) immunity (15, 26, 35, 36). Thus, it is unclear whether high temperatures impair the fish's ability to respond to an infection, especially under the thermal conditions that cultured fish such as Atlantic salmon may realistically face during the summer months; i.e., an incremental temperature increase to levels that approach or exceed their upper lethal limit (10, 13, 37).

Hypoxia has been associated with disease outbreaks (38–40), and is also known to adversely impact fish immune function. Rodriguez et al. (41) reported that acute hypoxia reduced the constitutive transcript levels of *ifna* and *mx* in the kidney of Atlantic salmon. Chronic and moderate intermittent hypoxia reduce leucocyte respiratory burst in this species (42). Finally, Kvamme et al. (43) exposed post-smolt Atlantic salmon to chronic hypoxia and then subjected them to a viral or bacterial immune-stimulation, and showed that while hypoxia reduced or delayed the expression of immune-related genes the overall effects were not large. However, Kvamme et al. (43) compared “control” fish kept at 74 ± 3.6% of air saturation with those kept at 52 ± 5.2% (mean ± SD) of air saturation, and the oxygen level that the “control” fish were exposed to is already considered hypoxic for this species.

Very few authors have examined the impact of the combination of moderate hypoxia and high temperatures on fish immune responses (44, 45), and no studies have investigated the impact of moderate hypoxia in combination with incremental increases in temperatures as high as 20°C on Atlantic salmon immune function; conditions that appear to be prevalent in Tasmania (10, 13) and led to mass mortalities at salmon sea-cage sites in Newfoundland in the summer of 2019 (46). Hence, in this study, Atlantic salmon were subjected to an incremental temperature increase (1°C week per week, from 12 to 20°C), then 4 weeks at 20°C, under normoxia or moderate hypoxia (~70% air saturation). Thereafter, the fish's innate immune function was evaluated prior to, and 6, 12, 24, and 48 h after, they were given an intraperitoneal (IP) injection of the multivalent vaccine Forte V II.

## Materials and Methods

This study was conducted in accordance with the guidelines of the Canadian Council on Animal Care, and approved by the Institutional Animal Care Committee of Memorial University of Newfoundland, Canada (Protocol ^#^16-90 KG). This research was conducted as part of the Mitigating the Impact of Climate-Related Challenges on Salmon Aquaculture (MICCSA) project, and the effects of these experimental protocols on growth characteristics (specific growth rate, food consumption, feed conversion ratio etc.) and mortality have recently been published (37).

### Animals

Atlantic salmon of Saint John River strain were obtained from Northern Harvest Sea Farms Ltd., and initially held at the Dr. Joe Brown Aquatic Research Building (JBARB; Ocean Sciences Centre, MUN) in 3,000 L circular tanks for 1 month. During this period, seawater temperature and oxygen levels were 10 ± 1°C and >95% of air saturation, respectively, photoperiod was 12 h light: 12 h dark, and the fish were fed a commercial salmonid diet (EWOS Dynamic S, EWOS Canada Ltd, British Columbia, Canada) at a ration of 1% body weight day^−1^. After acclimation, the fish were netted, lightly anesthetized in seawater containing tricaine methanesulfonate (AquaLife TMS, 0.1 g L^−1^; Syndel Laboratories Ltd, Nanaimo, BC, Canada), weighed, and had a PIT (Passive Integrated Transponder) tag implanted in their peritoneal cavity for individual identification. The fish were then returned to the same tanks for approximately 1 month.

### Experimental Design

A total of 360 salmon were moved from the JBARB, and randomly distributed into six 2200 L circular experimental tanks (60 fish tank^−1^) located in the Laboratory for Atlantic Salmon and Climate Change Research (LASCCR, Ocean Sciences Centre, St. John's, NL, Canada). After acclimating to the experimental tanks for 10 days, all fish were netted, lightly anesthetized (0.1 g L^−1^ TMS), and had their initial mass recorded (137.6 ± 1.3 g; mean ± SE). The fish were then allowed to recover for 15 days at 12 ± 1°C at an oxygen level of >95% air sat (photoperiod of 14 h light: 10 h dark). After this period, duplicate tanks were split into three treatments: control (CN), warm and normoxia (WN), and warm and hypoxia (WH). One header tank was designated to supply the control tanks, and the other header tank to supply the WN and WH tanks. For the control treatment, temperature, and oxygen levels were kept constant during the entire experiment at 12°C and >95% (air sat.), respectively. For the WH treatment, the oxygen level in the tanks was gradually lowered to 70% (air sat.) over a period of 2 weeks by reducing oxygenation in the header tank that fed these tanks, whereas oxygen levels in the WN treatment was maintained at 100–110% oxygen by placing oxygen diffusers in these tanks. Thereafter, the temperature in the WN and WH tanks was gradually increased from 12 to 20°C (by 1°C per week); the weekly temperature increments were 0.3°C (from days 1 to 3), 0.1°C on day 4, and then no change from days 5–7. When the water temperature in the WN and WH tanks reached 20°C, the fish were acclimated to these conditions for an additional 4 weeks. The salmon were carefully fed by hand twice daily to satiation (at 9:00 and 15:00) with commercial salmon feed (as described above), with feeding stopped when a few pellets began to accumulate on the bottom of each tank. See Supplementary Information in Gamperl et al. (37) for more specific information on these experimental protocols.

The tanks were supplied with seawater at 15 L min^−1^ (flow-through system) at the desired temperature. Seawater temperatures were controlled with the aid of large plate heat-exchangers that delivered heated seawater to a vacuum degasser that supplied each header tank, and tank temperatures and dissolved oxygen levels in all the experimental tanks were monitored every morning (YSI, ProODO, OH, USA). In addition, a Fibox 3 (LCD V3) meter with a pre-calibrated dipping probe (PreSens; Regensburg, Germany), and a computer running LCDPST3 Version 2.0.1.0 software (PreSens), were used to monitor dissolved oxygen levels in one of the hypoxic tanks continuously [see Gamperl et al. (37)]. Ammonia and nitrite levels in the tanks were also monitored (LaMotte test kit, Maryland, USA) on a weekly basis, and remained within acceptable levels (i.e., below 0.02 and 0.5 mg L^−1^, respectively).

After the WN and WH fish had been at 20°C for 4 weeks, eight fish per treatment (4 fish tank^−1^) were sampled (see below for details) for the assessment of basal (constitutive) immune function and head kidney transcript expression (i.e., at time 0). Two days after this initial sampling, the remaining fish in the tank were anesthetized in seawater containing 0.1 g L^−1^ TMS and then: (1) given an IP injection of Forte V II (Elanco Limited, Charlottetown, PEI, Canada; see additional information below) or phosphate-buffered-saline (PBS; sham-injected); and (2) injected with fluorescent elastomer (VIE; Northwest Marine Technology Inc., Ancortes, WA, USA) in the cartilage just behind the eye, with different colors for Forte V II (red) and PBS (yellow). This procedure allowed for the quick identification of PBS or Forte V II-fish in the tanks, and was chosen to minimize inflammation as this tissue is poorly vascularized. Following the IP and VIE procedures, the fish were returned to the same tank. Four fish (*N* = 8) per tank for each group (CN-PBS, CN-Forte V II; WN-PBS, WN-Forte V II; WH-PBS and WH-Forte V II) had their spleen and head kidney sampled at 6, 12, 24, and 48 h post-injection (HPI). At 24 HPI, blood was also collected by caudal puncture to measure respiratory burst, the hemolytic activity of the alternative pathway of the complement system and plasma lysozyme concentration, and the spleen was weighed to calculate spleen-somatic index. Finally, we measured head kidney transcript expression levels of 12 genes (see Table 1) involved in the salmon's anti-bacterial and/or anti-viral response in all groups at all time points.

**Table 1:** Primers used in the Real-time quantitative polymerase chain reaction (qPCR) analyses.

**Gene of interest**	**Nucleotide sequence (5^**′**^-3^**′**^)**	**^b^Efficiency (%)**	**Amplicon size (bp)**	**(GenBank Acc. No.)**	**References**
Interleukin 1 beta (*il1b*)	F: GTATCCCATCACCCCATCAC R: TTGAGCAGGTCCTTGTCCTT	99	119	AY617117	(47)
Interleukin 8a (*cxcl8*, alias *il8-a*)	F: GAAAGCAGACGAATTGGTAGAC R: GCTGTTGCTCAGAGTTGCAAT	104	99	BT046706	(47)
Cyclooxygenase-2 (*cox2*)	F: ACCTTTGTGCGAAACGCTAT R: GAGTAGGCCTCCCAGCTCTT	109	113	AY848944	(48)
Cathelicidin b (*camp-b*)	F: AGACTGGCAACACCCTCAAC R: TTGCCTCTTCTTGTCCGAAT	103	112	AY360357	(49)
Hepcidin a (*hamp-a*)	F: ATGAATCTGCCGATGCATTTC R: AATGGCTTTAGTGCTGGCAG	96	134	BT125319	(49)
Toll-like receptor 5a (soluble) (*stlr5-a*)	F: ATCGCCCTGCAGATTTTATG R: GAGCCCTCAGCGAGTTAAAG	96	103	AY628755	(50)
Interferon gamma (*ifng*)	F: CCGTACACCGATTGAGGACT R: GCGGCATTACTCCATCCTAA	103	133	AJ841811	(48)
Interferon regulatory factor 7b (*irf7-b*)	F: GTCAGTGGTAAAATCAACACGC R: CACCATCATGAAACGCTTGGT	91	99	FJ517644	(48)
Signal transducer and activator of transcription 1b (*stat1-b*)	F: GTTCAGGATGCAGAGCATGA R: CCATCCCATTCACCTCTTGT	99	109	BT048927	(48)
Radical S-adenosyl methionine domain containing protein 2b (*rsad2-b*, alias *viperin-b*)	F: TTCCTGGCATGGATAGGTGT R: CTTGGAGTTGTCGCTGGTTT	102	113	DY728694	Caballero-Solares et al. (in preparation)
Interferon-induced GTP-binding protein b (*mx-b*)	F: ACGCACCACTCTGGAGAAAT R: CTTCCATTTCCCGAACTCTG	94	184	BT044881	(48)
Interferon stimulated gene 15a (*isg15-a*)	F: AAAGTGGCCACAACAAAGCAG R: ATAGGAGCGGGCTCCGTAATC	90	140	BT049918	(48)
^a^Eukaryotic translation initiation factor 3 subunit D (*etif3d*)	F: CTCCTCCTCCTCGTCCTCTT R: GACCCCAACAAGCAAGTGAT	94	105	GE777139	(48)
^a^Polyadenylate-binding protein 1 (*pabpc1*)	F: TGACCGTCTCGGGTTTTTAG R: CCAAGGTGGATGAAGCTGTT	102	108	EG908498	(48)

a *Normalizers*.

b *Amplification efficiencies were calculated using a 5-point 1:3 dilution series starting with cDNA representing 10 ng of input total RNA. See methods for details*.

### Antigen Preparation and IP Injection

Forte V II was obtained in the form of a vaccine. This vaccine contains formalin-inactivated cultures of Infectious Salmon Anemia Virus (ISAV), *Aeromonas salmonicida, Vibrio anguillarum* serotypes I & II, *V. ordalii*, and *V. salmonicida* serotypes I & II in a liquid emulsion with an oil based adjuvant. The vaccine was allowed to warm to room temperature to facilitate injection; 50 μL was injected independent of fish mass, as recommended by the manufacturer. PBS was filtered using a 0.2 μm cartridge prior to injection, and administered using the same procedure as for Forte V II. Fish were fasted for 48 h prior to injection.

### Sampling

At time 0 (pre-injection) and at 6, 12, 24, and 48 HPI, the fish were quickly euthanized (in seawater containing 0.4 g L^−1^ TMS) and had their fork length and weight measured. The head kidney and spleen were then quickly removed, placed in separate 1.5 ml RNase-free tubes, snap-frozen in liquid nitrogen, and then stored at −80°C until RNA was prepared for the assessment of gene transcription. In addition, at 24 HPI, the spleens were weighed to measure the spleen-somatic index, and 2 mL of blood was collected by caudal puncture. For each individual, one hundred microliters of whole blood were immediately used in the respiratory burst assay. The remaining blood was centrifuged at 800 × g for 2 min at 4°C, and the plasma was transferred to a 1.5 ml tube and snap-frozen in liquid nitrogen before being stored at −80°C for the determination of hemolytic activity of the complement system and lysozyme concentration.

### Humoral Immune Response

#### Respiratory Burst

The production of reactive oxygen species (ROS) by salmon blood leukocytes was measured by chemiluminescence following the methods described by Marnila et al. (51), Nikoskelainen et al. (20) and Nikoskelainen et al. (52). Briefly, whole blood (2 μL mL^−1^ final concentration) was added to a mixture of 1 mM luminol (5-amino-2,3-dihydro 1,4-phthalazinedione, Sigma, St. Louis, MO, USA) in 0.2 M sodium borate and PBS. Then, 225 μL of this solution was added to triplicate wells of a 96-well white cliniplate (Cat. No. 28298-610, VWR International, Mississauga, ON), followed by 25 μL of 20 mg mL^−1^ zymosan from *Saccharomyces cerevisiae* (Sigma, Z4250). Thereafter, chemiluminescence was measured over 60 min using a microplate reader (SpectraMax M5; Molecular Devices, Sunnydale, CA, USA) at room temperature (~20°C). During this time, a curve of the luminescence counts per second (LCS) was obtained, and peak values were taken to represent the respiratory burst of each sample.

#### Hemolytic Activity of the Alternative Pathway of the Complement System

Complement hemolytic activity was measured according to Ferriani et al. (53) and Polhill et al. (54) with modifications made by Zanuzzo et al. (55) for salmonids. Initially, a sample of rabbit blood was mixed with an equal volume of Alsever solution (anticoagulant, pH 6.1). Then, an equal volume of TEA-EDTA chelating buffer (triethanolamine ethylenediamine tetraacetic acid; 0.1 M, pH 7.4 with 0.1% gelatin) was added, incubated for 15 min at 37°C, and then centrifuged at 800 × g for 10 min at 4°C to separate the cells [predominantly erythrocytes (i.e., red blood cells, RBCs)]. The rabbit RBCs (RaRBCs) were then washed three times in TEA-Mg^2+^ buffer (2 mM, pH 7.4) with successive centrifugations (at 800 × g for 10 min at 4°C), and stored for up to 15 days in Alsever solution at 4°C. Finally, before use, 1 mL of the RaRBC suspension was washed three times in TEA-EGTA-Mg^2+^ buffer [triethanolamine ethylene glycol tetraacetic acid (8 mM), with 2 mM of Mg^2+^ and 0.1% gelatin, pH 7.4], with successive centrifugations (at 800 × g for 10 min at 4°C), and then adjusted using the same buffer to an optical density of between 0.7 and 0.8 at 700 nm. The assay was carried out at a 1:8 dilution (75 μL of plasma combined with 125 μL of TEA-EGTA-Mg^2+^ buffer and 400 μL of the RaRBC suspension) and absorbance was measured for 20 min at 35°C. Plasma aliquots were heated for 30 min at 56°C to provide a negative control. Hemolytic complement activity for each sample was measured as the time (seconds) required for the initial optical density to be reduced by one-half (50% of RaRBC hemolysis by the complement alternative pathway).

#### Plasma Lysozyme Concentration

Plasma lysozyme concentration was determined according to Demers and Bayne (56). Briefly, standard solutions (0–10 ng μL^−1^) of hen egg white lysozyme (HEWL, Sigma-Aldrich, L6876) were prepared fresh from a frozen aliquot using 66 mM potassium phosphate buffer, pH 6.2. Then, dilutions of the standard and of serum (25 μL) were placed into triplicate wells of a 96-well plate. One hundred and seventy-five microliters of a 0.075% (wt:v) *Micrococcus lysodeikticus* (Sigma-Aldrich, M3770) suspension prepared in the same buffer were then added to each well. After rapid mixing, absorbance was measured at 450 nm every 20 s for 15 min at room temperature (~20°C) using a microplate reader (SpectraMax M5; Molecular Devices, Sunnydale, CA, USA). The rate of decrease in absorbance for each sample was then compared to that obtained with the standard curve, and lysozyme was expressed in ng μL^−1^ based on hen egg white lysozyme activity (ng μL^−1^ HEWL eq) which was used as the standard.

### Immune-Related Transcript Expression

#### RNA Preparation

Head kidney and spleen samples (~100 mg tissue per sample) were homogenized in 400 μL of TRIzol Reagent (Invitrogen) using individual RNase-free pestles and a motorized Kontes RNase-Free Pellet Pestle Grinder (Kimble Chase, Vineland, NJ). An additional 400 μL of TRIzol Reagent (Invitrogen) were then added to each sample, mixed by pipetting, and the homogenates frozen on dry ice and stored at −80°C. Frozen homogenates were further processed by slowly thawing them on ice and then passing them through a QIAshredder (QIAGEN, Mississauga, ON) spin column following the manufacturer's instructions. Two-hundred microliters of TRIzol (Invitrogen) were then added to each sample to make a total homogenate volume of ~1 mL, and the TRIzol total RNA extractions were then completed following the manufacturer's instructions.

Each TRIzol-extracted RNA sample (45 μg) was treated with 6.8 Kunitz units of DNaseI (RNase-Free DNase Set, QIAGEN) with the manufacturer's buffer (1x final concentration) at room temperature for 10 min to degrade any residual genomic DNA. DNase-treated RNA samples were then column-purified using the RNeasy Mini Kit (QIAGEN) following the manufacturer's instructions. RNA integrity was verified by 1% agarose gel electrophoresis, and RNA purity was assessed by A260/280 and A260/230 NanoDrop UV spectrophotometry for both the pre-cleaned and the column-purified RNA samples. Column-purified RNA samples had A260/280 ratios between 2.0 and 2.3, and A260/230 ratios between 1.9 and 2.3.

#### cDNA Synthesis and qPCR Parameters

First-strand cDNA templates for qPCR were synthesized in 20 μL reactions from 1 μg of DNaseI-treated, column-purified, total RNA using random primers (250 ng; Invitrogen), dNTPs (0.5 mM final concentration; Invitrogen) and M-MLV reverse transcriptase (200 U; Invitrogen) with the manufacturer's first strand buffer (1X final concentration) and DTT (10 mM final concentration) at 37°C for 50 min.

All PCR amplifications were performed in 13 μL reactions using 1X Power SYBR Green PCR Master Mix (Applied Biosystems), 50 nM of both the forward and reverse primers and cDNA representing 4 ng of input total RNA, and using the ViiA 7 Real Time PCR system (384-well format) (Applied Biosystems). The real-time analysis program consisted of 1 cycle of 50°C for 2 min, 1 cycle of 95°C for 10 min, and 40 cycles of 95°C for 15 s and 60°C for 1 min, with fluorescence detection at the end of each 60°C step, and was followed by dissociation curve analysis.

#### Endogenous Control (Normalizer) Selection

Transcript expression levels of the target genes [i.e., transcripts of interest (TOI)] were normalized to transcript expression levels of two endogenous control genes. These endogenous controls were selected from six candidate normalizers [*60S ribosomal protein L32* (*rp132;* BT043656), β*-actin* (*actb*; BG933897), *elongation factor 1-alpha 1* (*ef1a1*; AF321836), *elongation factor 1-alpha 2* (*ef1a2*; BT058669), *eukaryotic translation initiation factor 3 subunit D* (*etif3d*; GE777139) and *polyadenylate-binding protein 1* (*pabpc1*; EG908498)]. Normalizer testing was performed separately on 24 HPI and on 48 HPI samples. At 24 HPI, the fluorescence threshold cycle (C_T_) values of 12 head kidney and of 12 spleen samples from the control treatment [CN-PBS, CN-Forte V II (*N* = 6 per group)] were measured; at 48 HPI, the C_T_ values of 24 head kidney samples from each of the three treatments [6 groups: CN-PBS, CN-Forte V II; WN-PBS, WN-Forte V II; WH-PBS, WH-Forte V II (*N* = 4 per group)] were measured. These were analyzed separately using geNorm (57). Using this software, *etif3d* [geNorm M = 0.147 (24 HPI), 0.157 (48 HPI)] and *pabpc1* [geNorm M = 0.165 (24 HPI), 0.160 (48 HPI)] were determined to be stably expressed, and thus, were selected as the normalizers for both the pilot and main qPCR studies. When the experimental qPCR studies were performed, the endogenous control transcripts were indeed stably expressed in all samples (e.g., see Supplementary Table 1).

#### Real-Time Quantitative Polymerase Chain Reaction (qPCR)

As both the head kidney and spleen have immune-relevant functions, a preliminary study was conducted to determine which tissue would be best to assess changes in immune-relevant transcript expression levels. Briefly, transcript expression levels of 6 biomarker genes playing putative anti-bacterial and/or anti-viral roles [*interleukin 8a* (*cxcl8*, alias *il8-a*), *cyclooxygenase-2* (*cox2*)*, cathelicidin b* (*camp-b*)*, interferon gamma* (*ifng*)*, interferon regulatory factor 7b* (*irf7-b*), and *interferon stimulated gene 15a* (*isg15-a*)] were measured in the head kidney and spleen of the same salmon from the CN treatment at 24 HPI [i.e., CN-PBS and CN-Forte V II (*N* = 8 per group)]. On each plate, for every sample, the TOIs and endogenous controls were tested in triplicate and a no-template control was included. The relative quantity (RQ) of each transcript was determined using the ViiA 7 Software Relative Quantification Study Application (Version 1.2.3) (Applied Biosystems/Life Technologies), with normalization to both *etif3d* and *pabpc1* transcript levels, and with amplification efficiencies incorporated. For each TOI, the sample with the lowest normalized expression (mRNA) level, irrespective of tissue, was set as the calibrator sample (i.e., assigned an RQ value = 1.0).

Based on this preliminary experiment we selected the head kidney as the target tissue for the main qPCR study to assess how temperature/hypoxia affected the transcript expression levels of immune-relevant genes. While the transcript expression levels (i.e., PBS vs. Forte V II stimulated) obtained for the two tissues were similar, the data for the head kidney was less variable (i.e., fewer outliers were detected; 5 vs. 13 overall), and the expression of more genes was significantly different (see Supplementary Figure 1).

In the main qPCR study, transcript expression levels of 12 genes were assessed in head kidney samples from all groups in the study (see Experimental Design). More specifically, six of these genes are highly involved in the anti-bacterial response [*interleukin 1-beta* (*il1b*), *il8-a, cox2, camp-b, hepcidin a* (*hamp-a*), and *toll-like receptor 5a (soluble)* (*stlr5-a*)] (**Figure 2**), 3 in both the anti-bacterial and anti-viral responses [*ifng, irf7-b* and *signal transducer and activator of transcription 1b* (*stat1-b*)] (**Figure 3**), and 3 specifically in the anti-viral response [*radical S-adenosyl methionine domain containing protein 2b* (*rsad2-b*, alias *viperin-b*), *interferon-induced GTP-binding protein b* (*mx-b*) and *isg15-a*] (**Figure 4**). The sequences of the primer pairs used in the qPCR assays are presented in Table 1. All of these primers had been previously subjected to quality assurance testing (see references in Table 1), and primers for *viperin-b* were kindly provided by Dr. Albert Caballero-Solares (in preparation). On each plate, for every sample, the TOIs and endogenous controls were tested in triplicate, and a plate linker sample (i.e., a sample that was run on all plates in a given study; a transcript/plate was deemed acceptable only if its linker C_T_ value was within 0.5 cycles of the linker samples of all other plates in the study) and a no-template control were included. The relative quantity (RQ) of each transcript was determined as described above; for each TOI, the sample with the lowest normalized expression (mRNA) level was set as the calibrator sample (i.e., assigned an RQ value = 1.0).

### Statistical Analyses

Values were identified as outliers if they were > 4 or < −4 of the studentized x predicted y, and only four values were removed from the analyses. Some of the data were log_10_ transformed prior to statistical analysis as they failed normality (Cramer Von Mises) and/or homoscedasticity tests (Brown–Forsythe). One-way ANOVAs, followed by Duncan's new multiple range *post-hoc* tests were used to test for differences in constitutive (basal) transcript expression among the groups at 20°C. Two-way ANOVAs [3 (treatments) × 2 (injections; PBS, Forte V II)], followed by Duncan's new multiple range (MRT) *post-hoc* tests, were used to examine the effects of temperature and moderate hypoxia on respiratory burst, plasma lysozyme concentration, hemolytic activity of the complement system, and spleen-somatic index at 24 HPI. A three-way ANOVA factorial model [3 (treatments) × 2 (injections; PBS, Forte V II) × 4 (time points; 6, 12, 24, and 48 HPI)] would have led to too many interactions, and tested factors that were not related to the main hypotheses of this study. Thus, a two-way ANOVA considering only fish injected with Forte V II [3 (treatments) × 4 (sampling points; 6, 12, 24, and 48 h)], and a second two-way ANOVA considering only fish injected with PBS [3 (treatments) × 4 (time points; 6, 12, 24 and 48h)], followed by Duncan's new MRTs, were used to examine the effect of temperature and moderate hypoxia on transcript expression levels at the different time points post-injection. In addition, we used *t*-tests to compare the transcript expression levels between Forte V II and PBS-injected fish within each treatment and time point. Finally, within each injection group (i.e., PBS or Forte V II), Dunnett's tests were used to test whether values at 6, 12, 24, and 48 HPI were significantly different from “initial” (time 0) values. In all cases, *P* < 0.05 was used as the level of statistical significance. All data in the paper are expressed as mean ± SE. However, data presented in the Supplementary Figure 1 accompanying this paper are presented as mean ± SD to show the absolute variation.

## Results

### Humoral Immune Responses

**Figure 1:**
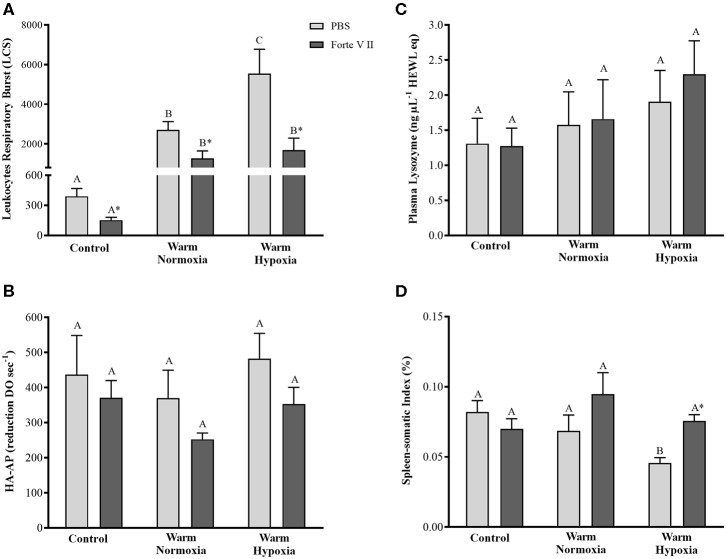
Respiratory burst **(A)**, hemolytic activity of the complement system **(B)**, serum concentration of lysozyme **(C)**, and spleen-somatic index **(D)** in salmon held at 12°C (under normoxia) or 20°C (under normoxia or mild hypoxia) (i.e., treatment) 24 h post-intraperitoneal injection with PBS or Forte V II (i.e., injection group). Different capital letters indicate a significant difference (*P* < 0.05) among treatments for PBS or Forte V II-injected fish. An asterisk (*) indicates a significant difference between PBS and Forte V II injection groups within a treatment. Values are means ± 1 standard error (S.E.), *N* = 7–8.

At 24 HPI, the leukocytes of PBS injected fish held at 20°C under normoxia or hypoxia had significantly higher (~ 6 and 14-fold, respectively) respiratory burst compared with CN (12°C) fish (Figure 1A). Furthermore, after the Forte V II injection, the leukocytes of WH fish had significantly higher (2-fold) respiratory burst compared with WN fish. In the CN fish, the injection of Forte V II resulted in a 61% decrease in respiratory burst (*P* < 0.05). This effect was also apparent in the WN and WH fish, where values were decreased by 53 and 69%, respectively, following V II injection (*P* < 0.05). There were no significant differences in the hemolytic activity of the complement system or plasma lysozyme concentrations among treatments or injection groups (Figures 1B,C). The spleen-somatic index of PBS-injected CN and WN fish was not different, but the SSI of the WH fish was ~30% lower compared with these other 2 treatments (Figure 1D). Although Forte V II injection did not affect the SSI in CN and WN fish, it increased SSI in the WH treatment to levels measured in the CN and WN treatments.

### Immune-Related Transcript Expression Levels

#### Effects of PBS and V II Injection in Control Fish

**Figure 2:**
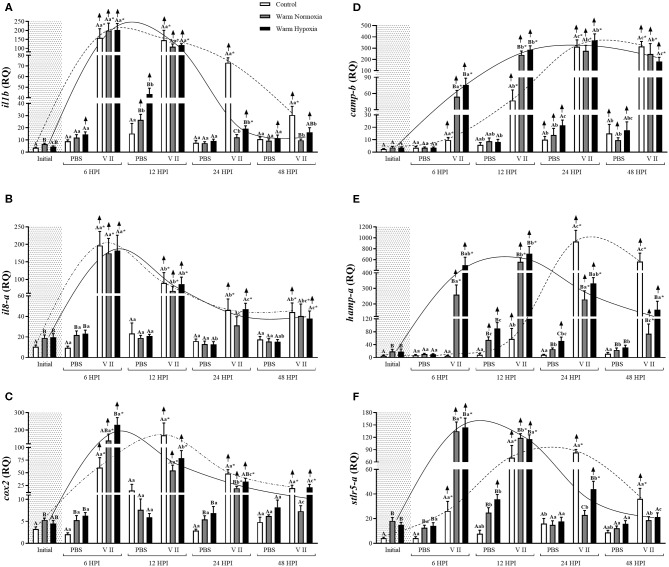
Transcript expression levels of 6 anti-bacterial biomarkers [*il1b* **(A)**, *il8-a* **(B)**, *cox2* **(C)**, *camp-b* **(D)**, *hamp-a* **(E)**, and *stlr5-a* **(F)**] in the head kidney of salmon held at 12°C (under normoxia) or 20°C (under normoxia or mild hypoxia) (i.e., treatment) 6, 12, 24, and 48 h post-intraperitoneal injection with PBS or Forte V II (i.e., group). Different capital letters indicate a significant difference (*P* < 0.05) among treatments within an injection group at a given time point (e.g., the three treatments six HPI after PBS injection) whereas dissimilar lower case letters indicate a significant difference over time within a treatment/group (e.g., control PBS-injected fish at 6, 12, 24, and 48 HPI). An asterisk (*) indicates a significant difference between PBS and Forte V II fish within a treatment at the same time point post-injection. The arrows indicate a significant difference compared with initial (constitutive expression) levels, within an injection group. Dotted (control) or solid lines (warm normoxia and hypoxia) illustrate the general pattern of changes in transcript expression levels for a given gene over time in Forte V II-injected fish, and are not based on any mathematical analysis. Transcript levels are presented as mean ± SE relative quantity (RQ) values (i.e., values for the transcript of interest were normalized to both *etif3d* and *pabpc1* transcript levels and were calibrated to the individual with the lowest normalized expression level of that given transcript). *N* = 7–8.

**Figure 3:**
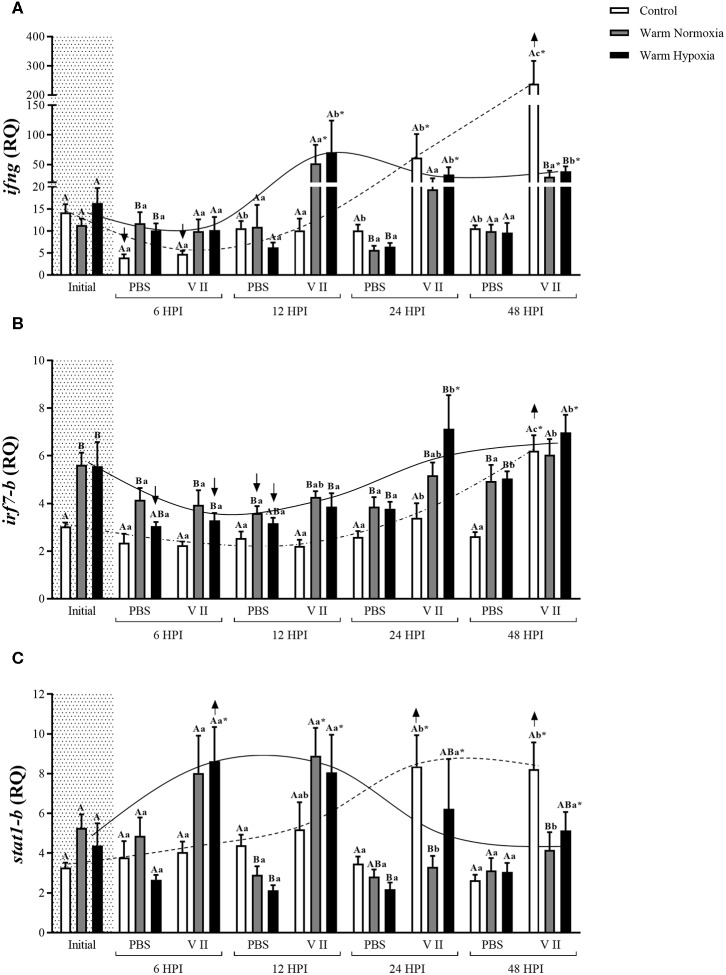
Transcript expression levels of three anti-bacterial/anti-viral biomarkers [*ifng* **(A)**, *irf7-b* **(B)**, and *stat1-b* **(C)**] in the head kidney of salmon held at 12°C (under normoxia) or 20°C (under normoxia or mild hypoxia) (i.e., treatment) 6, 12, 24, and 48 h post-intraperitoneal injection with PBS or Forte V II (i.e., injection group). Different capital letters indicate a significant difference (*P* < 0.05) among treatments within an injection group at a given time point (e.g., the three treatments 6 HPI after PBS injection) whereas dissimilar lower case letters indicate a significant difference over time within a treatment/group combination (e.g., control PBS-injected fish at 6, 12, 24, and 48 HPI). An asterisk (*) indicates a significant difference between PBS and Forte V II injection groups within a treatment at the same time point post-injection. The arrows (↑ or ↓) indicate a significant difference compared with initial (constitutive expression) levels, within an injection group. Dotted (control) or solid lines (warm normoxia and hypoxia) illustrate the general pattern of changes in transcript expression levels for a given gene over time in Forte V II-injected fish, and are not based on any mathematical analysis. Transcript levels are presented as mean ± SE relative quantity (RQ) values (i.e., values for the transcript of interest were normalized to both *etif3d* and *pabpc1* transcript levels and were calibrated to the individual with the lowest normalized expression level of that given transcript). *N* = 7–8.

**Figure 4:**
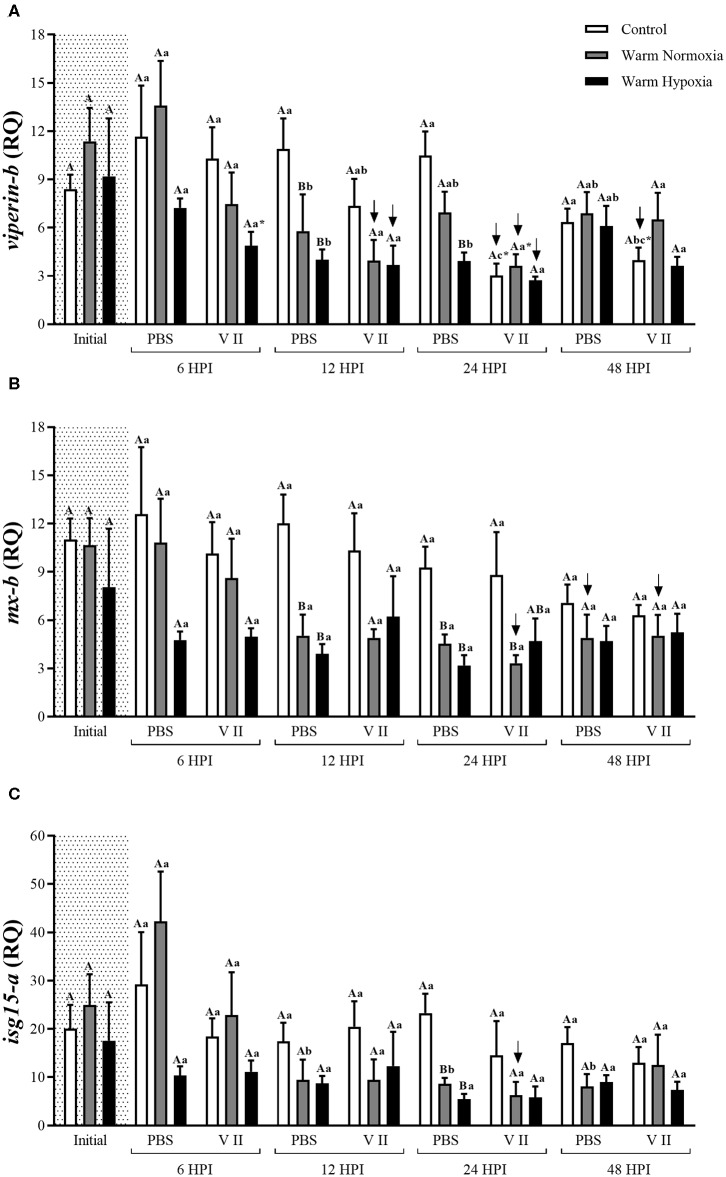
Transcript expression levels of 3 anti-viral biomarkers [*viperin-b* **(A)**, *mx-b* **(B)**, and *isg15-a* **(C)**] in the head kidney of salmon held at 12°C (under normoxia) or 20°C (under normoxia or mild hypoxia) (i.e., treatment) 6, 12, 24, and 48 h post-intraperitoneal injection with PBS or Forte V II (i.e., injection group). Different capital letters indicate a significant difference (*P* < 0.05) among treatments within an injection group at a given time point (e.g., the three treatments 6 HPI after PBS injection) whereas dissimilar lower case letters indicate a significant difference over time within a treatment/group combination (e.g., control PBS-treated at 6, 12, 24, and 48 HPI). An asterisk (*) indicates a significant difference between PBS and Forte V II injection groups within a treatment at the same time point post-injection. The arrows indicate a significant difference compared with initial (constitutive expression) levels, within an injection group. Transcript levels are presented as mean ± SE relative quantity (RQ) values (i.e., values for the transcript of interest were normalized to both *etif3d* and *pabpc1* transcript levels and were calibrated to the individual with the lowest normalized expression level of that given transcript). *N* = 7–8.

The injection of PBS only significantly affected the transcript expression levels of 2 of the 12 genes evaluated, and this effect was relatively small. CN (12°C; normoxia) fish injected with PBS had significantly higher transcript expression levels of *camp-b* at 24 and 48 HPI, while levels of *ifng* were significantly lower at 6 HPI compared with their respective baseline (pre-injection) values (Figures 2D, 3A, respectively). In contrast, V II injection significantly affected transcript expression levels of 10 of the 12 genes. Levels of 7 genes, including *il1b, il8-a, cox2, camp-b, hamp-a, stlr5-a*, and *ifng*, were greatly up-regulated by V II injection (i.e., by 44, 20, 53, 130, 167, 20, and 17-fold compared with constitutive values, respectively; Figures 2, 3A), whereas levels of *irf7-b*, and *stat1-b* only increased by 2 and 2.6-fold, respectively (Figures 3B,C). Interestingly, in CN fish injected with V II, transcript expression levels of *viperin-b* were significantly down-regulated at 24 (by 2.7-fold) and 48 HPI (by 1.5-fold) compared with PBS group (Figure 4A). The timing of peak expression also differed among the V II stimulated genes. Transcript expression levels of *il1b* and *il8-a* were highest (i.e., peaked) at 6 HPI, whereas those of *cox2* were highest at 12 HPI, those of *camp-b, hamp-a, stlr5-a*, and *stat1-b* were highest at 24 HPI, and those of *ifng* and *irf7-b* peaked at 48 HPI.

#### Effect of High Temperature on Immune-Related Transcript Expression

At the initial (constitutive) sampling, salmon acclimated to 20°C had significantly higher transcript expression levels of *il1b, il8-a, cox2, hamp-a, stlr5-a* (Figure 2) and *irf7-b* (Figure 3B) compared with CN (12°C) fish. However, post-injection transcript expression levels were very similar between CN and WN fish for most genes. For example, *il1b, il8-a, cox2, camp-b, irf7-b*, and *stat1-b* had very similar peak expression levels in both treatments (Figures 2, 3B,C). The exceptions were *hamp-a, ifng*, and *stlr5-a*. WN fish had lower maximum transcript levels of *hamp-a* (561 vs. 932 RQ, respectively) and *ifng* (52 vs. 239 RQ, respectively) compared with CN fish (Figures 2E, 3A). In contrast, WN fish had higher peak transcript expression levels of *stlr5-a* (~40%) compared with CN salmon (Figure 2F). Although the magnitude of post-injection transcript expression levels between CN and WN fish only differed for a few genes, the timing of peak expression for many of the genes was considerably earlier in the WN treatment compared with the CN treatment; e.g., while *cox2* transcript expression levels peaked at 6 HPI in WN fish, they peaked at 12 HPI in CN fish. Similar patterns were also observed for *hamp-a* (12 vs. 24 HPI; WN vs. CN, respectively), *camp-b* (12–24 vs. 24–48 HPI), *stlr5-a* (6 vs. 24 HPI), *ifng* (12 vs. 48 HPI), and *stat1-b* (6–12 vs. 24–48 HPI) (Figures 2, 3). In contrast, no differences were observed in the timing of peak transcript expression levels for *il1b*, and *il8-a* between WN and CN salmon (Figures 2A,B).

#### Effect of Moderate Hypoxia on Immune-Related Transcript Expression

There were no differences in the constitutive or maximum transcript expression levels between the WN and WH salmon (Figures 2–4). Furthermore, there were only a few differences in post-injection transcript expression levels between the WN and WH treatments. The WH group had higher post-injection transcript levels of *il1b* and *stlr5-a* at 24 HPI compared with WN fish (Figures 2A,F), whereas this same effect was observed for *cox2, hamp-a*, and *stat1-b* at 48 HPI (Figures 2C,E, 3C, respectively).

## Discussion

Climate change is predicted to increase water temperatures and decrease oxygen levels in the marine environment, and this may affect fish health and welfare, including their capacity to respond to pathogens (10, 13, 15, 39, 40). However, there is conflicting information about whether, and to what extent, these environmental factors may impact the immune defenses of fish (15). Thus, in this study, Atlantic salmon were subjected to a controlled incremental increase in water temperature (i.e., from 12 to 20°C, at a rate of 1°C per week) under normoxia or moderate hypoxia (~70% of air sat.) and then held at 20°C for 4 weeks. These conditions (i.e., an incremental temperature increase in association with moderate hypoxia) realistically reflect what farmed salmon face, or may face, in the future during the summer months (10–13). Thereafter, the salmon were immune-stimulated with the commercial vaccine Forte V II, and sampled at 6, 12, 24, and 48 HPI. We showed that while manipulating these environmental conditions did not affect the activity of complement system or plasma lysozyme concentration, high temperature (20°C) significantly increased respiratory burst and that the combination of this challenge with moderate hypoxia further increased this response (see PBS injection group, Figure 1). In addition, we report that high temperature up-regulated the constitutive expression of *il1b, il8-a, cox2, hamp-a, stlr5-a*, and *irf7-b* transcripts in the head kidney and hastened the increase in *cox2, hamp-a, camp-b, stlr5-a, ifng*, and *stat1-b* transcript expression levels post-stimulation with Forte V II. Interestingly, although high temperature affected the timing of changes in transcript expression levels, it did not have major effects on the magnitude of these changes, and hypoxia had few additional effects. Collectively, our results indicate that moderate hypoxia in combination with incremental increases in temperatures as high as 20°C do not impair the capacity of the Atlantic salmon's innate immune system to respond to bacterial pathogens.

### Impact of High Temperature, Hypoxia and Forte V II on the Humoral Immune Response and SSI

In our study, the respiratory burst of circulating (blood) leukocytes increased by ~ 6-fold in 20°C- compared with 12°C-acclimated fish, and the addition of moderate hypoxia induced a further increase in this response (Figure 1A). Nikoskelainen et al. (20) showed that respiratory burst increased in rainbow trout (*Oncorhynchus mykiss*) following long-term acclimation to high temperature (20 vs. 5°C). Further, it is well documented that long-term exposure to high temperatures increases the number of circulating leukocytes, and in particular the number of neutrophils in teleost species such as turbot (*Scophthalmun maximus*) (58), catfish (*Pangasianodon hypophthalmus*) (59) and Atlantic salmon (32). Hypoxia has also been shown to promote an increase in the number of leukocytes in rainbow trout (60). Thus, we hypothesize that exposure to high temperatures increased the number of circulating leukocytes, in particular neutrophils, and that hypoxia potentiated this response. Furthermore, the vaccine Forte V II significantly decreased respiratory burst, independent of temperature or oxygen level, and we attribute this reduction to the migration of circulating leukocytes into the abdominal cavity (i.e., the site of Forte V II injection). This hypothesis is supported by preliminary measurements using flow cytometry which showed that there was a reduction in the number of circulating leukocytes in fish injected with Forte V II compared with those injected with PBS (data not shown).

In our study, we did not observe differences in hemolytic activity of the complement system, in plasma lysozyme concentration or in the spleen-somatic index between fish exposed to high temperature alone or in combination with hypoxia compared with control fish (Figures 1B–D). In contrast, rainbow trout exposed to high temperature (20°C) for 57 days had an increased activity of the complement system (20), whereas no differences were observed in Atlantic cod (*Gadus morhua*) complement activity after long-term exposure to 1, 7, or 14°C (33). Valero et al. (61) investigated the effects of seasonal (slow) temperature changes on lysozyme concentration in European sea bass (*Dicentrarchus labrax*), and also found no impact of temperature. Finally, Salazar-Lugo et al. (62) did not find differences in the non-specific immune response of tambaqui (*Colossoma macropomum*) following 21 days of exposure to high temperatures. Thus, our results are consistent with the literature, and suggest that long-term acclimation to high temperatures does not impair the humoral immune responses of fish, and may even enhance these responses.

### Effects of High Temperature on Constitutive Transcript Expression Levels

In our study, fish acclimated to 20°C had higher constitutive expression of *il1b, il8-a, cox2, hamp-a, stlr5-a*, and *irf7-b* (Figures 2, 3B). These results agree with recent research conducted by our group which investigated the transcriptomic responses of salmon to 20°C acclimation using the 44-K Agilent salmon microarray and qPCR, and revealed that the constitutive expression of 15 important immune-related transcripts clustered very differently compared with control fish when subjected to principal component analysis (PCA) [Beemelmanns et al. (in preparation)]. The up-regulated transcripts observed at the initial sampling are related to a number of important aspects of the fish innate immune response including the inflammatory process (*il1b* and *il8*), prostaglandin production (*cox2*) and bacterial recognition (*stlr5-a*), or are antimicrobial peptides (*hamp-a*) or transcription regulatory factors (*irf7-b*) (63–69). Hyperthermia is known to induce inflammatory signaling in mammals, but the effects of high temperatures on inflammation in fish is still poorly understood (27). The increase in *il1b* expression observed in salmon acclimated to 20°C in this study is consistent with a similar study performed by Perez-Casanova et al. (34) on Atlantic cod, and has also been observed in mammals (70, 71). Collectively, these data suggest that IL-1β may play an important physiological role during prolonged heat exposure, and it could also be modulating the expression of genes encoding other inflammatory mediators such as *il8-a, cox2, hamp* and other antimicrobial peptides (63, 64, 72, 73).

Another plausible explanation for how long-term exposure to high temperatures could modulate the constitutive transcription of immune-related genes and inflammation is changes in the fish's bacterial community (74). Several studies show that temperature affects bacterial diversity in the water (75) as well as the fish gut microbiota (76–78), and a number of studies have linked gut microbiota load and diversity with the fish's immune system (79, 80). For example, gut microbiota can modulate the transcription of several immune-related genes in the digestive tract and also affect the innate immune response (81–83). Taken together, these findings suggest that changes in the profile of constitutive immune-related transcript expression could be partially related to inflammatory signaling as a direct effect of high temperature and/or an indirect effect through changes in the diversity and abundance of the fish's microbial community. Although, we have raised several plausible explanations of how long-term exposure to high temperature may modulate the constitutive transcription of immune-related genes, little information is available and this research area deserves further attention.

### Effects of Forte V II on the Anti-bacterial and Anti-viral Innate Immune Response

To our knowledge, no study has used the vaccine Forte V II, or injected similar bacterial and viral PAMPs (*A. salmonicida, V. anguillarum, V. ordalii*, and *V. salmonicida* and ISAV) concurrently. The Forte V II vaccine greatly increased the transcript expression levels of anti-bacterial genes such as *il1b, il8-a, cox2, camp-b, hamp-a, stlr5-a, infg*, and *stat1-b*, in the head-kidney of Atlantic salmon (Figures 2, 3A,C). The up-regulation of these genes is consistent with studies that have injected teleost fishes with *A. salmonicida* or *Vibrio* sp. For example, STAT1 is a signal transduction protein critical to the interferon pathway, and has been shown to be up-regulated upon *A. salmonicida* infection in Japanese eel (*Anguilla japonica*) (84). The IP-injection of formalin-killed *A. salmonicida* induced a similar increase in the expression of *il1b, camp-b* and *il8-a* at 24 HPI in the spleen of trout acclimated at 10°C (85). Finally, Atlantic salmon experimentally infected with *V. salmonicida* showed a similar up-regulation of *il1b, il8-a*, and *stlr5-a* expression in the spleen between 6 and 12 HPI (86).

Although we observed a robust anti-bacterial response, the vaccine Forte V II which contains formalin-killed cultures of ISAV did not stimulate the expression of transcripts that are specifically related to the anti-viral response (i.e., *viperin-b, mx-b, and isg15-a*; Figure 4). ISAV is one of the most important viruses impacting marine salmonid aquaculture, and disease outbreaks have been reported in several regions of the world, but mainly Norway, Canada and Chile (87). With respect to post-injection effects of ISAV vaccines on innate immune gene expression, Collet et al. (88) IP injected salmon at 10°C with a commercial ISAV vaccine, and showed that the expression of *mx* in the blood had a sustained increase starting at 8 days post-injection (DPI). McBeath et al. (89) reported that the expression levels of *mx* and type I and II *ifn* in the head kidney increased drastically after day 4, and peaked at day 6, post-infection with ISAV in salmon acclimated at 11°C. Wolf et al. (90) found that an IP administered ISAV vaccine up-regulated *mx* transcript levels in the spleen after 8 DPI. LeBlanc et al. (91) used a 32 k cDNA microarray platform and reported that no transcripts were dysregulated at 24 HPI and that only 25 transcripts were ≥2-fold differentially expressed at 3 DPI. Further, LeBlanc et al. (92) injected salmon at 13°C with ISAV and observed that the expression of *mx* and *stat1* only started to be up-regulated at 10 and 20 DPI, respectively, and Caruffo et al. (93) reported that ISAV infection (IP) only induced the transcription of *mx* in the head-kidney of salmon after 40 DPI. These studies suggest that a relatively long time is necessary for the salmon immune system to respond to ISAV, and that there might have been up-regulation of anti-viral transcripts in our study if we had sampled for a longer period.

Interestingly, we observed that the transcript expression of some specific anti-viral immune genes such as *viperin-b, mx-b* and *isg15-a*, was down-regulated in fish injected with Forte V II compared with the PBS group or their constitutive levels (see Figure 4). According to Eberl (74), and the concept of “immunity by equilibrium,” the activation of one type of response may inhibit another type, and an immune equilibrium is maintained by the competing immune responses. Boucontet et al. (94) used zebrafish (*Danio rerio*) to test whether viral infection (with Sindbis virus) would interfere with bacterial susceptibility, and whether bacterial infection (with *Shigella flexneri*) would affect the susceptibility to viral infection. The authors found that virus-infected fish were hyper-susceptible to bacterial infection, while fish infected with bacteria first and virus later did not experience increased mortality or microbial burden. These results are remarkably similar to what has been observed in mouse models (95) and in humans (96). Although we still do not know if the mechanisms that lead to increased bacterial or viral susceptibility upon infection with other types of pathogen-associated antigens are shared between fish and mammals, our results suggest that a robust anti-bacterial response may suppress anti-viral transcript expression in fish, and could have contributed to the absence of up-regulated anti-viral transcript expression levels in this study. This finding also suggests that concomitant vaccination for bacterial and viral pathogens might not be as effective as if they are administered separately (i.e., at different times), at least with respect to non-specific immune responses. The hypothesis that fishes have a defined and limited capacity to respond to antigenic substances is supported by Busch (97).

### Effects of High Temperature on Anti-bacterial Transcript Expression

The effects of temperature on fish biology have been extensively studied. However, information about how long-term acclimation to high temperatures affects the innate immune response, specifically the expression of immune-related genes, is still limited. The majority of studies in this area suggest that high temperatures induce an earlier expression of immune-relevant genes (14, 98). However, studies have often compared immune-related gene expression in fish held at different temperatures at the same time point (25) or only at a limited number of time points (99); which could potentially have led to questionable conclusions. In addition, many studies in fish have only investigated the effects of short-term exposure to high temperature on immune-related gene expression (29, 44, 98, 100, 101). In this study, water temperature was slowly increased from 12 to 20°C under conditions of normoxia and moderate hypoxia [i.e., mimicking what salmon at cage-sites may face during the summer months (10–12, 37)], and the salmon were sampled at 6, 12, 24, and 48 HPI with Forte V II or PBS. We found that high temperature hastened the peak in transcript expression levels of 5 genes (*cox2, camp-b, hamp-a, stlr5-a*, and *stat1-b*; Figures 2, 3C), but that the maximum post-injection levels were similar for eight of the nine anti-bacterial genes (Figures 2, 3). Taken together, our results indicate that temperatures up to 20°C hasten the induction, but do not alter the magnitude of, immune-related transcript expression levels in salmon. An earlier increase in expression levels of immune-related genes at high temperatures was also observed by Thanasaksiri et al. (102) for Japanese flounder (*Paralichthys olivaceus*) injected with polyinosinic:polycytidylic acid [poly (I:C)] at 25 vs. 15°C, and by Hori et al. (103) in Atlantic cod injected with formalin-killed *A. salmonicida* at 16 vs. 10°C. Similarly, *mx* and *stat1* transcripts were strongly up-regulated in Japanese flounder injected with poly (I:C) at 25°C at 3 HPI, whereas at 15°C, the genes were only up-regulated at 24 HPI (98). Furthermore, the magnitude and timing of expression observed in our study are consistent with data obtained by a number of other authors: for *tnfa1, tnfa2, il1b*, and *il8* in peripheral blood leukocytes and head kidney homogenates of southern Bluefin tuna (*Thunnus thynnus*) stimulated with LPS at 25 and 18°C (27); by Raida and Buchmann (31) when measuring the expression of *il6, il10, ifng, tnfa*, and *tgf* in the spleen of rainbow trout acclimated for 8 weeks at 25 or 15°C and bath vaccinated with *Y. ruckeri*; and as shown by Kaneshige et al. (104) in Japanese flounder injected with formalin-killed *Edwarsiella tarda* at 25 and 15°C for *il1b, hamp*, and *ifng*.

Although we measured similar increases in transcript expression levels in the 12 and 20°C groups for almost all genes examined, the expression of *ifng* did not follow this pattern and it had lower peak expression (by approx. 4.5-fold) in fish acclimated to 20°C compared with CN fish acclimated to 12°C (Figure 3A). This result is in agreement with Kaneshige et al. (104) who observed lower peak *irf-1* transcript expression in the head kidney of Japanese flounder (*Paralichthys olivaceus*) acclimated to high temperature (22 vs. 15°C). These authors suggested that certain genes involved in the IFN-γ signaling pathway might be sensitive to high temperatures and that this leads to increased susceptibility to pathogen infection. Wang et al. (99) also found that the transcript level of *ifng* was inhibited at low and high temperatures (21 and 33°C) compared with optimal temperatures (such as 25 and 29°C) after Nile tilapia (*Oreochromis niloticus*) were vaccinated with inactivated *Streptococcus agalactiae*. Although we did not find a difference in the maximal post-injection expression of *stat1-b*, which is one of the transcription factors activated by type II IFNs such as IFN-γ (105), these findings suggest that the resistance to pathogens afforded by the IFN-γ signaling pathway may be reduced at higher temperatures. Similarly, vaccines that are dependent on type II IFN stimulation might not be as effective at warmer water temperatures. Overall, these data suggest that *ifng* may potentially be a useful biomarker for evaluating the impact of high temperatures on the immune responses of fish.

### Effects of Hypoxia on Immune-Related Transcript Expression

In this study, we also examined whether the combination of moderate hypoxia and an incremental temperature increase to 20°C would impair the innate immune response as such conditions have been observed in salmon sea-cages in Tasmania (10), Norway (12) and Newfoundland (11). Further, these conditions recently led to massive mortalities at several cage-sites in Newfoundland during the summer of 2019 (46). While the addition of moderate hypoxia did not affect peak post-vaccination transcript expression, we did observe that fish exposed to an incremental temperature increase and moderate hypoxia had higher expression levels of *i1*β and *stlr5-a* at 24 HPI compared with fish only exposed to high temperature, and that a similar profile was apparent for *cox2, hamp-a* and *stat1-b* at 48 HPI (Figures 2, 3C). Collectively, these data suggest that hypoxia increases the duration of elevated immune-relevant transcript expression when fish are exposed to high temperatures, and this finding is consistent with the results of Kvamme et al. (43). These authors injected salmon with poly (I:C) or a water-based *V. anguillarum* vaccine at 10°C, and reported that there was a more rapid down-regulation of gene transcription in fish acclimated to 75 vs. 52% air saturation.

### The Overall Effects of an Incremental Temperature Increase and Hypoxia on Innate Immune Responses

In summary, we found that an incremental increase to 20°C enhanced leukocyte respiratory burst, increased the constitutive expression of several immune-related genes, hastened the peak in transcript expression levels after Forte V II injection, but inhibited the expression of *ifng* post-stimulation. However, these impacts were relatively small overall, and our findings indicate that an incremental temperature increase up to 20°C [a temperature only 2–3°C below the acclimation temperature where Atlantic salmon mortalities become significant (37, 106)] modulates, but does not have a major effect on, the salmon's innate immune response. This conclusion is consistent with several studies on a variety of teleost species (25, 34, 58, 61). However, there are also a number of studies that have shown that short-term (acute) exposure to high temperatures impairs or limits the immune response (19, 29, 30, 101, 107, 108). There is little information on the effect of the rate of temperature change (increase) on fish immune responses (15), and although the current study did not examine this issue, we suspect that the non-deleterious effect of long-term exposure to high temperature may, in part, be related to circulating cortisol levels in fish exposed to long-term vs. acute increases in temperature. Plasma cortisol has been shown to increase sharply after an acute exposure to high temperature in fish (109–112), while chronic exposure to high temperature revealed few impacts on the stress physiology/cortisol levels in Atlantic salmon (113) or other teleost species (25, 34, 114, 115). In fact, we measured resting plasma cortisol levels in a very similar experiment to this one [see Experiment ^#^2 in Gamperl et al. (37)] and they did not become elevated until the fish experienced a temperature of 22°C [Zanuzzo et al. (in preparation)]. This hormone is a well-known modulator of the immune system, and higher cortisol levels limit or reduce the immune response [e.g., see Zanuzzo et al. (116)]. Perez-Casanova et al. (34) suggested that when exposed to an incremental temperature increase, immune function is influenced by complex interactions between thermal effects and temperature-induced stress (i.e., elevated circulating cortisol levels). Thus, the effects of faster changes in water temperature (e.g., degrees per hour) on the fish's immune response might not directly reflect a temperature effect, but rather the effect of stress (elevated cortisol levels). Such an effect could largely explain the conflicting results surrounding the effect of high temperature on the fish immune response to bacterial and/or viral antigens.

Few studies have examined the effect of chronic hypoxia on the innate immune response of fish, but those that have suggest that this environmental condition may impair the capacity of fish to respond to pathogens. For example, chronic and moderate intermittent hypoxia have been shown to decrease leucocyte respiratory burst in Atlantic salmon (42). Gallage et al. (117) observed a lower, and delayed, antibody production in Nile tilapia chronically exposed to hypoxia, and the authors associated the delay in antibody production with a reduction in the transcript levels of *i1*β, *tcr*β, and *mhcII*β. Kvamme et al. (43) found that Atlantic salmon exposed to chronic hypoxia had either reduced expression or a delay in the expression of immune-related genes. Even fewer studies have investigated the impact of the combination of hypoxia and high temperature on fish immune function. However, Wang et al. (44) reported that hypoxia reduced the protection afforded by vaccination in Nile tilapia independent of acclimation temperature (30 vs. 35°C). Further, Niklasson et al. (45) exposed salmon to chronic hypoxia (50% air sat.) at 8 and 16°C, and showed that hypoxia negatively affected the intestinal mucosal immune system and that these effects were more pronounced at 16°C; these data suggest that hypoxia has additive adverse effect to those of high temperature. Although, the addition of moderate hypoxia potentially represents an additional challenge to that of high temperature, contrary to our expectation, it had few effects on the salmon's innate immune function (with the exception of enhancing respiratory burst in PBS-injected fish and delaying the return of transcript expression to pre-vaccination levels). The limited effects of chronic hypoxia on the innate immune responses in this study may be related to the level of hypoxia used (~70% of air saturation), which might have enabled a suite of physiological responses that allowed WH fish to up-regulate immune-related transcript expression to levels similar to WN and CN fish. This hypothesis does have some support in the literature. Moderate hypoxia (60–65% air sat.) had no effect on the severity or progress of pancreas disease in Atlantic salmon infected with Salmonid Alphavirus (SAV) (118). Magnoni et al. (119) exposed rainbow trout to 4.5 mg L^−1^ of dissolved oxygen for 49 days, but reported no changes in the lysozyme, peroxidase and alternative complement pathway activities, and concluded that the trout displayed an adaptive response to the level of chronic hypoxia to which they were exposed.

## Summary and Perspectives

Due to accelerated climate change, fish will likely face incremental temperature increases that approach or exceed their upper thermal limit, and often in combination with hypoxia, in the future (1, 2, 7, 13). However, little is known about the impact of rising temperatures and reduced dissolved oxygen levels on fish immune defenses. In our study, we found that an incremental increase in water temperature to 20°C alone, or in combination with moderate hypoxia (70% air saturation), had only very limited impacts on the Atlantic salmon's capacity to mount an innate immune response. These findings do not support the hypothesis that salmon innate immune responses are greatly impaired at high temperature and hypoxia, and thus, suggest that such an effect is not responsible for disease outbreaks during the summer months. However, it is also very likely that the conditions examined in this study [i.e., 20°C and moderate hypoxia (70% air saturation] are on the edge/limit of the physiological tolerance for this strain of salmon [see Gamperl et al. (37)], and that further deterioration in these environmental conditions could lead to the impairment of salmon immune function.

Although this study examined the salmon's capacity (innate immunity) to respond to antigens from several pathogens, we cannot draw conclusions about the susceptibility to disease under different environmental conditions because we injected fish with formalin inactivated cultures of bacteria and / ISAV (i.e., commercial multivalent vaccine Forte V II). The susceptibility to disease at each temperature is specific, and a trade-off between the effects of temperature on the host and pathogen. For example, while high temperature (15 vs. 12°C) increases infection prevalence and pathogen burden in rainbow trout exposed to *Tetracapsuloides bryosalmonae* (28), it provides olive flounder (*Paralichthys olivaceus*) with enhanced protection against haemorrhagic septicaemia virus (i.e., no mortality at 20°C compared with 24% at 15°C) (24). In the latter study, the authors concluded that fish at lower temperature had a delay in the host response and that this provided a window for viral growth. In addition to susceptibility, the adaptive immune system might respond differently to temperature and hypoxia compared with the innate immune response. For example, Atlantic salmon vaccinated for *V. salmonicida* at 2, 4, and 6°C had better protection against infection than fish vaccinated at 8 or 10°C (120). Collectively, these findings suggest that we have a lot more to learn about fish immune function, and how to protect commercially important aquaculture species from pathogens in the face of accelerated climate change.

## Data Availability Statement

The qPCR data generated for this study are included in the article/Supplementary Material.

## Ethics Statement

This study was conducted in accordance with the guidelines of the Canadian Council on Animal Care, and approved by the Institutional Animal Care Committee of Memorial University of Newfoundland, Canada (Protocol ^#^16-90 KG).

## Author Contributions

Specifically, FZ, AG, AB, and MR contributed to the experimental design. FZ, AB, and AG carried out the experiment and samplings. JH performed the immune-related transcript expression study, and along with FZ wrote the materials and methods section. FZ performed the humoral immune assays, generated the figures, analyzed results and wrote the manuscript. AB, AG, JH, and MR revised the manuscript. All authors have made a substantial and intellectual contribution to the study, and approved it for publication.

## Conflict of Interest

The authors declare that the research was conducted in the absence of any commercial or financial relationships that could be construed as a potential conflict of interest.
