# Supplementary material for: The Innate Immune Response of Atlantic Salmon (Salmo salar) Is Not Negatively Affected by High Temperature and Moderate Hypoxia
Source: Front Immunol. 2020 May 27;11:1009. doi: 10.3389/fimmu.2020.01009 (PMC7268921; doi:10.3389/fimmu.2020.01009)
Supplement: Supplementary file 1 [file Data_Sheet_1.docx]

**Supplemental Table 1a** **|** Mean C_T_ values for the transcript of interest and the endogenous controls (*etif3d* and *pabpc1*). Numbers in the table are mean values ± 1 S.E.

|  | | **Study 1**^a^ | | | | | **Study 2**^a^ | | | | |
| --- | --- | --- | --- | --- | --- | --- | --- | --- | --- | --- | --- |
| **TOIs / EC (C_T_)** | | *irf7-b* | *isg15-a* | *mx-b* | *etif3d* | *pabpc1* | *stat1-b* | *stlr5-a* | *viperin-b* | *etif3d* | *pabpc1* |
| **Group** | |  |  |  |  |  |  |  |  |  |  |
| **Initial** | **CN** | 28.87 ± 0.18 | 30.48 ± 1.09 | 27.97 ± 0.50 | 22.28 ± 0.45 | 20.43 ± 0.25 | 28.97 ± 0.28 | 30.04 ± 0.75 | 28.44 ± 0.42 | 22.17 ± 0.17 | 20.47 ± 0.27 |
|  | **WN** | 27.95 ± 0.40 | 30.12 ± 1.05 | 28.09 ± 0.76 | 22.15 ± 0.16 | 20.51 ± 0.23 | 28.39 ± 0.58 | 27.92 ± 0.68 | 28.12 ± 0.75 | 22.15 ± 0.25 | 20.52 ± 0.20 |
|  | **WH** | 28.10 ± 0.53 | 31.44 ± 1.88 | 29.19 ± 1.34 | 22.15 ± 0.24 | 20.52 ± 0.18 | 28.87 ± 1.04 | 28.14 ± 0.69 | 28.76 ± 1.04 | 22.12 ± 0.29 | 20.57 ± 0.27 |
|  |  |  |  |  |  |  |  |  |  |  |  |
| **6 HPI**  **PBS** | **CN** | 29.42 ± 0.69 | 30.26 ± 1.57 | 28.27 ± 1.26 | 22.25 ± 0.26 | 20.44 ± 0.18 | 28.89 ± 0.83 | 30.26 ± 1.24 | 28.16 ± 0.96 | 22.19 ± 0.27 | 20.37 ± 0.23 |
|  | **WN** | 28.35 ± 0.60 | 29.55 ± 1.67 | 28.24 ± 1.16 | 22.17 ± 0.24 | 20.29 ± 0.37 | 28.57 ± 1.02 | 28.25 ± 0.54 | 27.80 ± 0.85 | 22.11 ± 0.23 | 20.26 ± 0.34 |
|  | **WH** | 28.76 ± 0.24 | 31.40 ± 1.32 | 29.14 ± 0.55 | 22.18 ± 0.19 | 20.27 ± 0.21 | 29.33 ± 0.52 | 28.13 ± 0.71 | 28.47 ± 0.44 | 22.11 ± 0.20 | 20.24 ± 0.24 |
| **6 HPI**  **V II** | **CN** | 29.53 ± 0.38 | 30.69 ± 0.98 | 28.41 ± 0.93 | 22.45 ± 0.23 | 20.55 ± 0.19 | 28.82 ± 0.73 | 27.84 ± 1.62 | 28.36 ± 0.95 | 22.36 ± 0.23 | 20.51 ± 0.18 |
|  | **WN** | 28.47 ± 0.72 | 30.64 ± 1.75 | 28.61 ± 1.33 | 22.10 ± 0.09 | 20.29 ± 0.15 | 27.74 ± 0.91 | 24.80 ± 0.96 | 28.71 ± 0.97 | 22.07 ± 0.10 | 20.27 ± 0.17 |
|  | **WH** | 28.51 ± 0.36 | 31.08 ± 1.02 | 28.92 ± 0.46 | 21.96 ± 0.20 | 20.14 ± 0.27 | 27.49 ± 0.82 | 24.50 ± 0.75 | 29.05 ± 0.85 | 21.93 ± 0.23 | 20.12 ± 0.35 |
|  |  |  |  |  |  |  |  |  |  |  |  |
| **12 HPI**  **PBS** | **CN** | 29.25 ± 0.44 | 30.72 ± 1.11 | 27.97 ± 0.80 | 22.28 ± 0.21 | 20.48 ± 0.19 | 28.67 ± 0.63 | 29.72 ± 1.54 | 28.19 ± 0.82 | 22.30 ± 0.23 | 20.47 ± 0.22 |
|  | **WN** | 28.54 ± 0.35 | 31.85 ± 1.32 | 29.26 ± 0.84 | 22.11 ± 0.14 | 20.33 ± 0.12 | 29.07 ± 0.50 | 27.23 ± 0.66 | 29.18 ± 0.96 | 22.08 ± 0.13 | 20.31 ± 0.13 |
|  | **WH** | 28.68 ± 0.43 | 31.51 ± 0.98 | 29.50 ± 0.91 | 22.06 ± 0.18 | 20.29 ± 0.16 | 29.40 ± 0.57 | 26.76 ± 0.71 | 29.39 ± 0.81 | 22.10 ± 0.32 | 20.21 ± 0.23 |
| **12 HPI**  **V II** | **CN** | 29.29 ± 0.58 | 30.48 ± 1.47 | 28.20 ± 1.12 | 22.07 ± 0.17 | 20.79 ± 1.18 | 28.39 ± 1.03 | 26.92 ± 2.47 | 28.64 ± 0.92 | 22.04 ± 0.17 | 20.29 ± 0.24 |
|  | **WN** | 28.24 ± 0.20 | 31.84 ± 1.32 | 29.09 ± 0.51 | 22.07 ± 0.13 | 20.33 ± 0.11 | 27.50 ± 0.62 | 24.86 ± 0.34 | 29.66 ± 0.96 | 22.08 ± 0.14 | 20.34 ± 0.11 |
|  | **WH** | 28.51 ± 0.52 | 32.25 ± 1.91 | 29.44 ± 1.48 | 22.08 ± 0.21 | 20.41 ± 0.21 | 27.77 ± 0.79 | 24.95 ± 0.40 | 30.04 ± 1.28 | 22.05 ± 0.17 | 20.39 ± 0.22 |
|  |  |  |  |  |  |  |  |  |  |  |  |
| **24 HPI**  **PBS** | **CN** | 29.32 ± 0.39 | 30.28 ± 0.81 | 28.43 ± 0.60 | 22.84 ± 1.23 | 20.60 ± 0.18 | 29.05 ± 0.49 | 28.47 ± 1.12 | 28.31 ± 0.77 | 22.32 ± 0.28 | 20.58 ± 0.22 |
|  | **WN** | 28.56 ± 0.53 | 31.60 ± 0.70 | 29.34 ± 0.56 | 22.16 ± 0.19 | 20.45 ± 0.12 | 29.25 ± 0.50 | 28.24 ± 0.88 | 28.85 ± 0.73 | 22.17 ± 0.18 | 20.46 ± 0.11 |
|  | **WH** | 28.55 ± 0.41 | 32.43 ± 0.83 | 29.96 ± 0.80 | 22.15 ± 0.21 | 20.44 ± 0.24 | 29.63 ± 0.56 | 27.91 ± 0.76 | 29.57 ± 0.56 | 22.13 ± 0.21 | 20.46 ± 0.24 |
| **24 HPI**  **V II** | **CN** | 28.81 ± 0.55 | 31.63 ± 1.55 | 28.69 ± 1.03 | 22.11 ± 0.12 | 20.37 ± 0.17 | 27.82 ± 0.85 | 25.78 ± 1.21 | 30.00 ± 0.72 | 22.09 ± 0.19 | 20.32 ± 0.18 |
|  | **WN** | 28.07 ± 0.57 | 32.68 ± 1.56 | 29.81 ± 0.58 | 22.13 ± 0.30 | 20.44 ± 0.24 | 28.99 ± 0.69 | 27.52 ± 0.95 | 29.70 ± 0.72 | 22.13 ± 0.34 | 20.41 ± 0.27 |
|  | **WH** | 27.53 ± 0.54 | 32.32 ± 0.90 | 29.34 ± 0.91 | 21.96 ± 0.31 | 20.30 ± 0.28 | 28.25 ± 0.91 | 26.29 ± 0.53 | 29.79 ± 0.36 | 21.91 ± 0.32 | 20.28 ± 0.30 |
|  |  |  |  |  |  |  |  |  |  |  |  |
| **48 HPI**  **PBS** | **CN** | 29.01 ± 0.29 | 30.57 ± 0.94 | 28.63 ± 0.79 | 22.10 ± 0.11 | 20.34 ± 0.12 | 29.32 ± 0.56 | 28.51 ± 0.75 | 28.76 ± 0.57 | 22.09 ± 0.14 | 20.36 ± 0.15 |
|  | **WN** | 28.08 ± 0.52 | 32.05 ± 1.60 | 29.41 ± 1.19 | 22.07 ± 0.15 | 20.35 ± 0.20 | 29.01 ± 0.81 | 28.24 ± 0.53 | 28.74 ± 1.06 | 22.02 ± 0.16 | 20.30 ± 0.19 |
|  | **WH** | 28.08 ± 0.34 | 31.62 ± 0.82 | 29.36 ± 0.82 | 22.10 ± 0.19 | 20.46 ± 0.22 | 29.12 ± 0.61 | 28.02 ± 0.67 | 29.12 ± 1.05 | 22.12 ± 0.18 | 20.47 ± 0.23 |
| **48 HPI**  **V II** | **CN** | 27.63 ± 0.58 | 30.88 ± 0.89 | 28.61 ± 0.53 | 21.93 ± 0.20 | 20.31 ± 0.28 | 27.66 ± 0.82 | 26.79 ± 1.01 | 29.43 ± 0.82 | 22.00 ± 0.23 | 20.33 ± 0.25 |
|  | **WN** | 27.69 ± 0.26 | 31.73 ± 1.51 | 29.22 ± 0.81 | 21.91 ± 0.20 | 20.35 ± 0.29 | 28.62 ± 0.61 | 27.65 ± 0.59 | 29.21 ± 1.01 | 21.9 ± 0.20 | 20.35 ± 0.30 |
|  | **WH** | 27.64 ± 0.44 | 32.04 ± 1.00 | 29.28 ± 0.87 | 22.14 ± 0.27 | 20.48 ± 0.26 | 28.43 ± 0.84 | 27.63 ± 0.82 | 29.68 ± 0.58 | 22.11 ± 0.29 | 20.49 ± 0.28 |

^a^For each qPCR study, 3 transcripts of interest (TOIs) and two endogenous controls (EC; *etif3d* and *pabpc1*) were run on each linked plate (n=9).

**Supplemental Table 1b** **|** Mean C_T_ values for the transcript of interest and the endogenous controls (*etif3d* and *pabpc1*). Numbers in the table are mean values ± 1 S.E.

|  | | **Study 3**^a^ | | | | | **Study 4**^a^ | | | | |
| --- | --- | --- | --- | --- | --- | --- | --- | --- | --- | --- | --- |
| **TOIs / EC (C_T_)** | | *camp-b* | *cox2* | *hamp-a* | *etif3d* | *pabpc1* | *il1b* | *il8-a* | *ifng* | *etif3d* | *pabpc1* |
| **Group** | |  |  |  |  |  |  |  |  |  |  |
| **Initial** | **CN** | 27.48 ± 0.62 | 33.03 ± 0.62 | 31.37 ± 0.81 | 22.39 ± 1.42 | 20.12 ± 0.17 | 29.35 ± 0.79 | 29.52 ± 0.68 | 29.65 ± 0.50 | 22.12 ± 0.25 | 20.30 ± 0.22 |
|  | **WN** | 27.02 ± 1.03 | 32.03 ± 0.49 | 29.68 ± 1.14 | 21.78 ± 0.12 | 20.06 ± 0.07 | 28.36 ± 0.56 | 28.70 ± 0.77 | 29.99 ± 0.64 | 21.99 ± 0.15 | 20.29 ± 0.10 |
|  | **WH** | 26.83 ± 0.53 | 32.46 ± 0.59 | 29.85 ± 1.11 | 21.77 ± 0.15 | 20.12 ± 0.15 | 29.01 ± 0.55 | 28.71 ± 0.67 | 29.60 ± 0.87 | 22.02 ± 0.20 | 20.40 ± 0.12 |
|  |  |  |  |  |  |  |  |  |  |  |  |
| **6 HPI PBS** | **CN** | 27.24 ± 0.93 | 33.27 ± 0.69 | 31.22 ± 1.10 | 22.41 ± 1.48 | 20.13 ± 0.18 | 28.00 ± 0.84 | 29.70 ± 0.67 | 31.62 ± 0.87 | 22.13 ± 0.25 | 20.29 ± 0.18 |
|  | **WN** | 26.80 ± 0.42 | 32.15 ± 0.84 | 30.14 ± 0.94 | 22.57 ± 2.13 | 19.89 ± 0.34 | 27.47 ± 0.56 | 28.42 ± 0.69 | 29.99 ± 0.79 | 22.01 ± 0.25 | 20.15 ± 0.32 |
|  | **WH** | 26.91 ± 0.72 | 31.83 ± 0.37 | 30.42 ± 0.86 | 21.84 ± 0.15 | 19.96 ± 0.20 | 27.19 ± 0.73 | 28.31 ± 0.56 | 30.04 ± 0.64 | 22.02 ± 0.18 | 20.18 ± 0.21 |
| **6 HPI**  **V II** | **CN** | 25.79 ± 1.18 | 29.36 ± 1.56 | 31.59 ± 1.24 | 22.03 ± 0.17 | 20.42 ± 0.76 | 24.45 ± 1.74 | 26.37 ± 2.20 | 31.41 ± 0.73 | 22.29 ± 0.20 | 20.45 ± 0.16 |
|  | **WN** | 23.34 ± 1.33 | 28.18 ± 1.72 | 26.23 ± 2.23 | 22.05 ± 0.55 | 20.04 ± 0.15 | 23.54 ± 1.07 | 25.70 ± 1.11 | 30.44 ± 1.35 | 21.99 ± 0.11 | 20.18 ± 0.17 |
|  | **WH** | 22.48 ± 0.77 | 26.95 ± 0.72 | 25.14 ± 2.11 | 21.77 ± 0.23 | 19.90 ± 0.30 | 23.23 ± 0.59 | 25.44 ± 0.78 | 30.32 ± 1.36 | 21.86 ± 0.18 | 20.06 ± 0.26 |
|  |  |  |  |  |  |  |  |  |  |  |  |
| **12 HPI PBS** | **CN** | 26.74 ± 1.30 | 32.56 ± 1.82 | 31.28 ± 1.44 | 22.03 ± 0.15 | 20.21 ± 0.14 | 28.02 ± 1.56 | 28.89 ± 1.13 | 30.16 ± 0.60 | 22.19 ± 0.20 | 20.33 ± 0.16 |
|  | **WN** | 25.86 ± 1.07 | 31.80 ± 0.96 | 27.88 ± 0.86 | 21.81 ± 0.10 | 20.04 ± 0.12 | 26.34 ± 0.78 | 28.56 ± 0.48 | 30.44 ± 1.10 | 21.99 ± 0.09 | 20.21 ± 0.12 |
|  | **WH** | 25.88 ± 1.08 | 31.88 ± 0.54 | 27.10 ± 0.81 | 21.80 ± 0.14 | 19.98 ± 0.11 | 25.48 ± 0.59 | 28.34 ± 0.38 | 30.79 ± 1.00 | 21.94 ± 0.15 | 20.16 ± 0.15 |
| **12 HPI**  **V II** | **CN** | 24.28 ± 2.04 | 29.13 ± 2.89 | 28.56 ± 3.15 | 21.90 ± 0.14 | 20.11 ± 0.18 | 25.00 ± 2.34 | 27.11 ± 1.69 | 30.37 ± 1.12 | 21.99 ± 0.18 | 20.22 ± 0.25 |
|  | **WN** | 20.95 ± 0.57 | 29.01 ± 0.63 | 24.38 ± 0.53 | 21.84 ± 0.17 | 20.10 ± 0.14 | 24.37 ± 0.52 | 26.89 ± 0.75 | 28.81 ± 1.71 | 21.96 ± 0.17 | 20.19 ± 0.21 |
|  | **WH** | 20.62 ± 0.53 | 28.51 ± 0.80 | 24.07 ± 0.86 | 21.76 ± 0.20 | 20.09 ± 0.12 | 24.03 ± 0.34 | 26.52 ± 0.66 | 28.81 ± 1.90 | 21.91 ± 0.16 | 20.25 ± 0.17 |
|  |  |  |  |  |  |  |  |  |  |  |  |
| **24 HPI PBS** | **CN** | 25.90 ± 1.05 | 33.06 ± 0.56 | 30.81 ± 0.68 | 22.11 ± 0.18 | 20.30 ± 0.16 | 28.53 ± 0.91 | 29.12 ± 0.56 | 30.33 ± 0.49 | 22.29 ± 0.18 | 20.49 ± 0.18 |
|  | **WN** | 25.60 ± 1.43 | 32.12 ± 0.60 | 29.13 ± 0.88 | 22.02 ± 0.29 | 20.16 ± 0.10 | 28.29 ± 0.77 | 29.41 ± 1.02 | 30.95 ± 0.65 | 22.09 ± 0.15 | 20.27 ± 0.11 |
|  | **WH** | 24.52 ± 0.78 | 31.86 ± 0.58 | 28.18 ± 0.98 | 21.91 ± 0.10 | 20.19 ± 0.16 | 28.00 ± 0.76 | 29.32 ± 0.65 | 30.90 ± 0.61 | 22.10 ± 0.16 | 20.36 ± 0.19 |
| **24 HPI**  **V II** | **CN** | 21.29 ± 1.95 | 29.72 ± 1.63 | 24.35 ± 1.79 | 21.92 ± 0.19 | 20.12 ± 0.12 | 25.27 ± 1.28 | 27.69 ± 0.86 | 28.85 ± 1.68 | 22.10 ± 0.16 | 20.31 ± 0.15 |
|  | **WN** | 20.87 ± 0.75 | 30.71 ± 1.15 | 26.02 ± 1.08 | 21.89 ± 0.18 | 20.16 ± 0.14 | 27.55 ± 0.91 | 28.26 ± 1.26 | 29.88 ± 1.65 | 22.06 ± 0.26 | 20.27 ± 0.18 |
|  | **WH** | 20.27 ± 0.67 | 29.71 ± 0.81 | 25.08 ± 0.73 | 21.71 ± 0.18 | 20.01 ± 0.15 | 26.68 ± 0.42 | 27.26 ± 0.39 | 28.26 ± 2.13 | 21.91 ± 0.24 | 20.19 ± 0.23 |
|  |  |  |  |  |  |  |  |  |  |  |  |
| **48 HPI PBS** | **CN** | 25.41 ± 1.39 | 32.35 ± 0.67 | 30.40 ± 1.00 | 21.81 ± 0.12 | 20.02 ± 0.08 | 27.63 ± 0.59 | 29.19 ± 1.48 | 29.96 ± 0.34 | 22.01 ± 0.13 | 20.19 ± 0.05 |
|  | **WN** | 25.59 ± 0.74 | 31.95 ± 0.33 | 29.27 ± 0.82 | 21.85 ± 0.16 | 20.11 ± 0.21 | 27.83 ± 0.51 | 28.96 ± 0.73 | 30.19 ± 0.53 | 22.03 ± 0.17 | 20.24 ± 0.20 |
|  | **WH** | 25.06 ± 1.20 | 31.80 ± 0.96 | 28.97 ± 1.00 | 21.83 ± 0.14 | 20.16 ± 0.19 | 27.74 ± 0.80 | 28.97 ± 0.70 | 30.47 ± 1.10 | 22.01 ± 0.15 | 20.30 ± 0.19 |
| **48 HPI**  **V II** | **CN** | 20.44 ± 0.65 | 30.70 ± 1.46 | 24.60 ± 1.35 | 21.64 ± 0.19 | 20.02 ± 0.24 | 26.20 ± 1.17 | 27.40 ± 0.84 | 26.12 ± 1.73 | 21.81 ± 0.22 | 20.16 ± 0.27 |
|  | **WN** | 21.70 ± 1.92 | 31.64 ± 0.56 | 27.86 ± 1.22 | 21.68 ± 0.12 | 20.05 ± 0.24 | 27.64 ± 0.59 | 27.88 ± 0.94 | 28.75 ± 0.80 | 21.82 ± 0.15 | 20.19 ± 0.26 |
|  | **WH** | 21.50 ± 0.68 | 30.63 ± 1.14 | 26.74 ± 1.43 | 21.87 ± 0.19 | 20.19 ± 0.22 | 27.38 ± 1.29 | 27.77 ± 0.73 | 28.39 ± 0.82 | 22.06 ± 0.23 | 20.37 ± 0.23 |

^a^For each qPCR study, 3 transcripts of interest (TOIs) and two endogenous controls (EC; *etif3d* and *pabpc1*) were run on each linked plate (n=9).

**
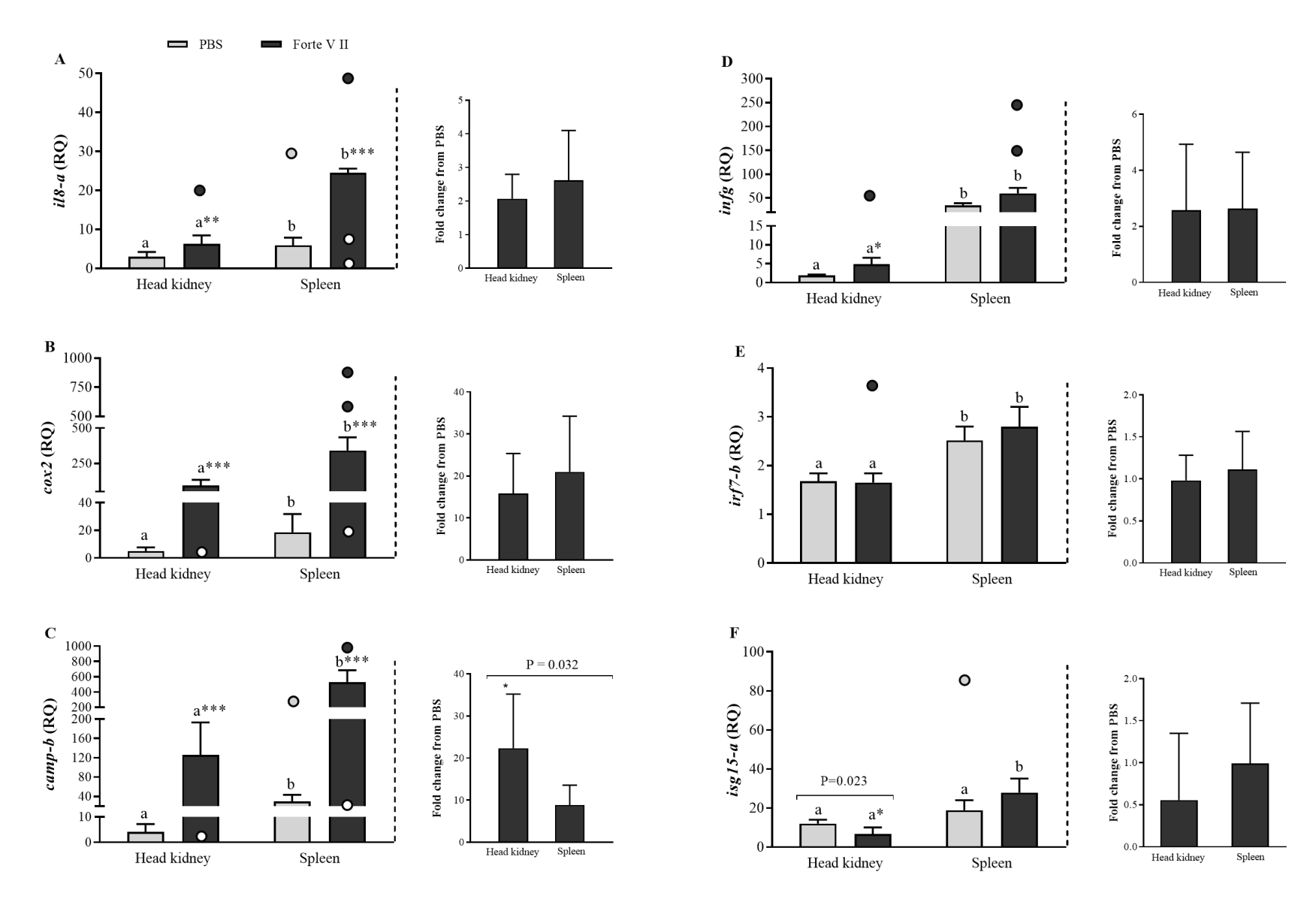
**

**Supplemental Figure 1 |** Head kidney and spleen transcript expression levels for 6 biomarker genes playing putative anti-bacterial and/or anti-viral roles in salmon held at 12°C (control treatment), 24 h post-injection with PBS or Forte V II. Different letters indicate a significant difference (P < 0.05) between head kidney and spleen within an injection group (PBS or Forte V II). Asterisks indicate a significant difference between PBS and Forte V II within a tissue (*P < 0.05; **P < 0.01; ***P < 0.001). Expression levels are presented as mean ± SD relative quantity (RQ) values (i.e., values for the transcript of interest were normalized to both *etif3d* and *pabpc1* transcript levels and were calibrated to the sample with the lowest normalized expression level of that given transcript, irrespective of tissue). N = 5-8. Numbers less than 8 indicate that values were removed based on a statistical test for outliers (studentized residual > 3 or < -3, SAS), and these outliers are represented by dots in each respective group. Data were analyzed using a two-way ANOVA (tissue vs. injection group), followed by Tukey’s post-hoc tests; t-tests were performed to assess transcript expression levels in Forte V II- vs. PBS-injected fish in the head kidney and spleen.
